# Supplementary material for: Splice-Junction-Based Mapping of Alternative Isoforms in the Human Proteome
Source: Cell Rep. Author manuscript; Available in PMC 2020 Jan 15. (PMC6961840; doi:10.1016/j.celrep.2019.11.026)
Supplement: 3 [file NIHMS1546469-supplement-3.zip › DF2/PXD0010154/Ovary-16-P04233-HSLEQKPTDAPPKESLELEDPSSGLGVTK.pdf]

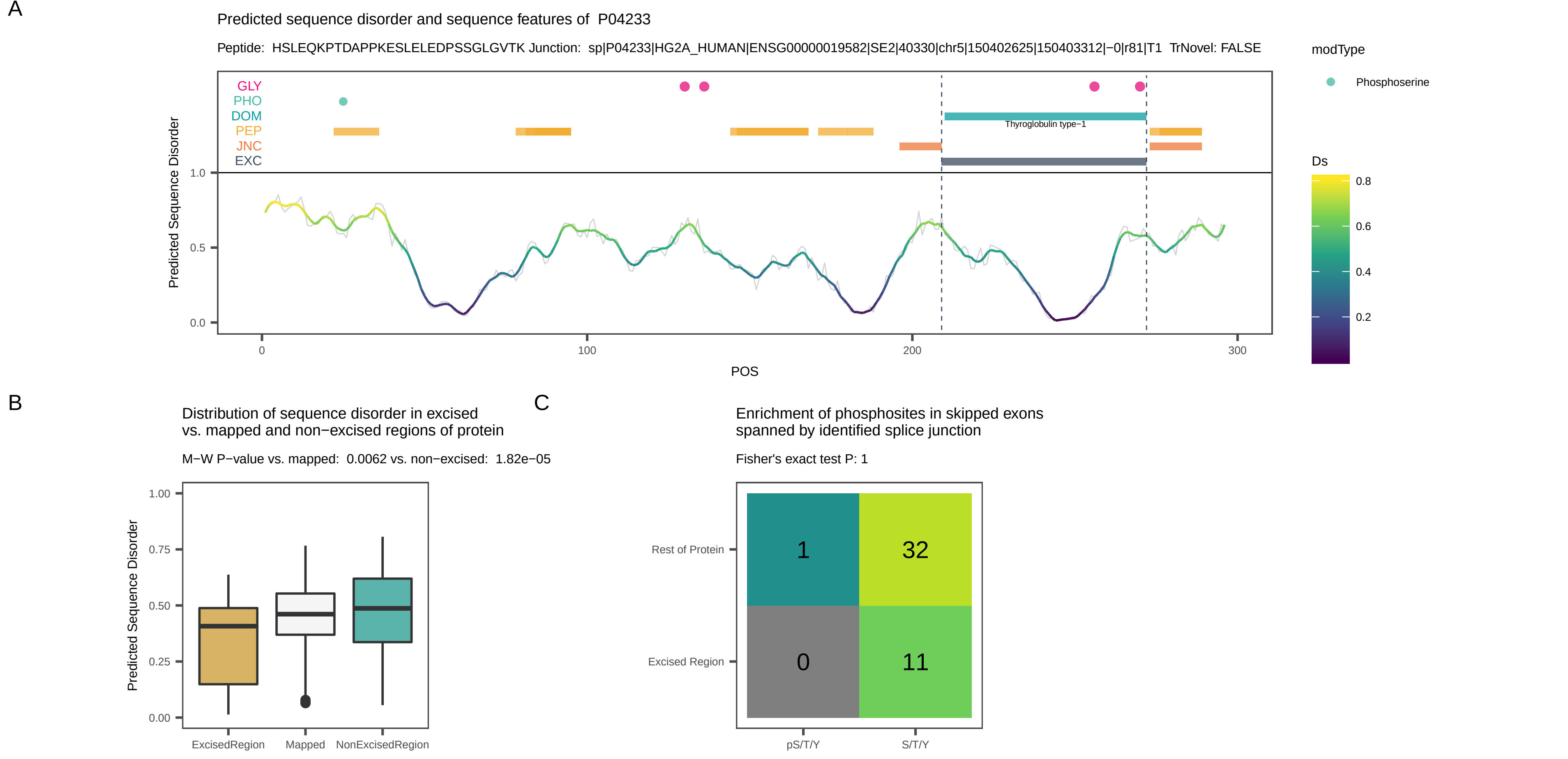
A
Predicted sequence disorder and sequence features of P04233
Peptide: HSLEQKPTDAPPKESLELEDPSSGLGVTK Junction: sp|P04233|HG2A_HUMAN|ENSG00000019582|SE2|40330|chr5|150402625|150403312|−0|r81|T1 TrNovel: FALSE
GLY
PHO
DOM
PEP
JNC
EXC
Thyroglobulin type−1
Predicted Sequence Disorder
1.0
0.5
0.0
0
100
200
300
POS
modType
Phosphoserine
Ds
0.8
0.6
0.4
0.2
B
Distribution of sequence disorder in excised
vs. mapped and non−excised regions of protein
M−W P−value vs. mapped: 0.0062 vs. non−excised: 1.82e−05
Predicted Sequence Disorder
1.00
0.75
0.50
0.25
0.00
ExcisedRegion
Mapped
NonExcisedRegion
C
Enrichment of phosphosites in skipped exons
spanned by identified splice junction
Fisher's exact test P: 1
Rest of Protein
Excised Region
1
32
0
11
pS/T/Y
S/T/Y
